# Self-Assembly and DNA Binding of the Blocking Factor in X Chromosome Inactivation

**DOI:** 10.1371/journal.pcbi.0030210

**Published:** 2007-11-09

**Authors:** Mario Nicodemi, Antonella Prisco

**Affiliations:** 1 Complexity Science and Department of Physics, University of Warwick, Warwick, United Kingdom; 2 Istituto Nazionale di Fisica Nucleare (INFN), Naples, Italy; 3 Institute of Genetics and Biophysics (IGB) “A. Buzzati Traverso” (ABT), CNR, Naples, Italy; Universitá di Torino, Italy

## Abstract

X chromosome inactivation (XCI) is the phenomenon occurring in female mammals whereby dosage compensation of X-linked genes is obtained by transcriptional silencing of one of their two X chromosomes, randomly chosen during early embryo development. The earliest steps of random X-inactivation, involving counting of the X chromosomes and choice of the active and inactive X, are still not understood. To explain “counting and choice,” the longstanding hypothesis is that a molecular complex, a “blocking factor” (BF), exists. The BF is present in a single copy and can randomly bind to just one X per cell which is protected from inactivation, as the second X is inactivated by default. In such a picture, the missing crucial step is to explain how the molecular complex is self-assembled, why only one is formed, and how it binds only one X. We answer these questions within the framework of a schematic Statistical Physics model, investigated by Monte Carlo computer simulations. We show that a single complex is assembled as a result of a thermodynamic process relying on a phase transition occurring in the system which spontaneously breaks the symmetry between the X's. We discuss, then, the BF interaction with X chromosomes. The thermodynamics of the mechanism that directs the two chromosomes to opposite fates could be, thus, clarified. The insights on the self-assembling and X binding properties of the BF are used to derive a quantitative scenario of biological implications describing current experimental evidences on “counting and choice.”

## Author Summary

In mammals, female cells silence one of their two X chromosomes to equalize X products with respect to males. The mechanism whereby cells count their X's and randomly choose the one to inactivate is, though, one of the most mysterious aspects of X chromosome inactivation (XCI). The longstanding hypothesis is that a molecular complex, a “blocking factor” (BF), exists: the BF is present in a single copy and can randomly bind to just one X per cell which is protected from inactivation, as the second X is inactivated by default. We add here a missing crucial step to such a picture: we explain, on a thermodynamic ground, why only one complex is formed in the cell, how it is self-assembled and how it selectively binds DNA recognition sequences. Such a process, leading to the spontaneous breaking of the binding symmetry of two equivalent targets, results from collective behavior at a molecular level whose general features are independent from the ultimate biochemical molecular details. It embodies, thus, a new general stochastic regulatory mechanism which could be relevant to a broad class of cell processes involving a random switch.

## Introduction

In diploid cells, most genes are expressed from both alleles; the most notable exception to this rule is X-linked genes in female mammals. During embryo development, one X chromosome, randomly selected, is transcriptionally silenced in female cells, so that the levels of X-derived transcripts are equalized in XX females and XY males [1]. The important scientific and medical implications of X chromosome inactivation (XCI) have focused substantial attention on the underlying molecular mechanisms [2–5]. Nevertheless, the nature of the signals that direct two identical X chromosomes to two opposite fates is still mysterious. Although XCI is one of the best-studied cases, it is estimated that about 10% of the total of our genes displays random monoallelic expression [6,7]. The understanding of the mechanisms that regulate this stochastic process is relevant, thus well beyond XCI.

The complete phenomenon leading to X inactivation involves several steps: counting of X chromosomes in the cell, choice of the inactive X, initiation and spreading of silencing on the designated inactive X, and maintenance of the inactive status through subsequent cell divisions [2–4]. As many aspects of initiation, spreading, and maintenance of X-inactivation are known [2–4], the very starting mechanism whereby cells count their X chromosomes and choose between two equivalent X is not understood, and especially surprising in random X inactivation of placental mammalian embryonic cells.

On the X, the DNA segment controlling silencing is the X-chromosome–inactivation center (*Xic*), containing several genes and regulators involved in XCI [2,3]. The *Xic* includes, in particular, the *Xist* (X inactive-specific transcript) gene that encodes a large noncoding RNA which is directly responsible for silencing by coating the presumptive inactive X. In the cells of a developing female embryo, before random X inactivation initiates, *Xist* is expressed at low levels from both *Xic*. Then in each cell *Xist* expression is upregulated on the future inactive X and silenced on the active X. Silencing of other genes on the *Xist*-expressing X chromosome follows rapidly. The X-linked regions involved in counting and choice map within the *Xic*, too (see [2,3,8–11] and references therein). Regions on autosomes (nonsexual chromosomes) have been also discovered that affect XCI [12]. Interestingly, diploid cells with X chromosome aneuploidy have only one active X, independently of the number of X chromosomes [2,3,13].

The basic observations listed above ground the hypothesis that “controlling factors” for counting and choice derive from autosomes and interact with cis-acting regulatory sequences on the X chromosomes. Some models explain counting and random choice in XCI [2,3] by assuming the existence of a “blocking factor” (BF), a complex made of autosomal (and X) factors binding to just one *Xic* per cell [14,15], which is protected from inactivation, as the second unprotected X in a female is inactivated by default by *Xist* coating. Models with more than one factor have been proposed as well [8,11,16]. It has been pointed out, however, that such an elegant picture cannot explain some recent experiments we discuss later on: e.g., the behavior of a set of homozygous XX deletions [8,17], or the discovery at the onset of XCI of X colocalization, specifically in the *Xic* region, a process necessary to attain proper XCI [18,19].

The nature of the BF (and its binding site on the X) is still unclear: it might be a unique nuclear component, such as an attachment site on the membrane, though it is mostly assumed [2,3] to be a diffusing molecule, such as a single protein or RNA, or a supermolecular complex. The idea that the BF is a single molecule capable, thus, to bind to a single *Xic*, is however unconvincing. How are autosomes regulated to produce precisely one molecule? When diffusing in the nucleus, its chances to miss the target are too high; in its travel to the target, the molecule could be subject, as well, to any sort of attack, from chemical modifications to degradations. Thus, the most convincing hypothesis about the identity of the BF is that it is a single complex formed by many autosomally (or X) derived molecules. This hypothesis raises, however, an important question: it is necessary to explain why only one factor is formed.

We study here a Statistical Mechanics quantitative version of the “BF” theory of X inactivation to answer such a question in the light of the above recent experiments. Within the framework of such a model, called the “symmetry breaking” (SB) model [20], we describe by use of thermodynamics how the “BF” complex is self-assembled and why only one is formed, i.e., how the symmetry in the binding of the complex to the equivalent X chromosomes is broken. We then consider the binding of the “BF” to DNA sequences of the future active X. Finally, we discuss deletions or insertions experiments, where a skewing in the binding energy to the two X is introduced, and we outline the effects on random XCI. Our model is investigated by Monte Carlo computer simulations and compared to experimental results.

## Results

### The Model

**Figure 1:**
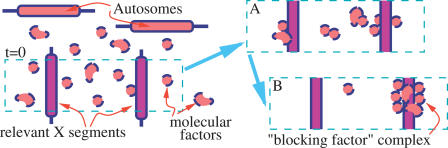
A Schematic Picture of Our Model A random initial configuration of molecular factors, deriving from autosomes, surrounds two parallel and equally binding X chromosome segments relevant to XCI. Molecules have a reciprocal affinity, *E*_0_, and can bind each other. As times goes on, particles form clusters (A), and if *E*_0_ is larger than a given threshold value *E* ^*^ (a value of the order of a weak hydrogen bond, see text) clusters coalesce into a single major complex attached to only one of the chromosomes (B).

In our model we consider the relevant proximal portions of the two *Xic*, where the BF binds, and an initially random distribution of diffusible molecules originated by autosomes in the surrounding space (see Figure 1). We assume that the molecular factors have a reciprocal affinity, i.e., they interact with each other, as a complex should be assembled.

**Figure 2:**
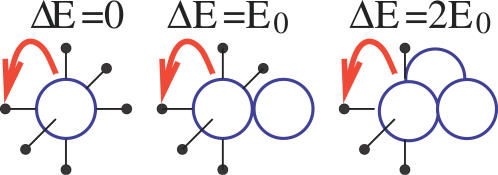
In Our Lattice Model Particles Interact with Those on Nearest Neighbor Vertexes, via an Effective Energy *E*_0_ In the left picture, no energy barrier has to be crossed for the particle to move to its left neighbor (Δ*E* = 0). In the central picture, the particle breaks one bond and the barrier is Δ*E* = *E*_0_; in the right picture Δ*E* = 2*E*_0_, since the moving particle has two neighbors.

In our schematic description, we consider a simple geometric configuration where the two X segments are parallel, at a given distance *L* in some units *d*_0_ (of the order of the molecule size), in a volume of linear sizes *L_x_* = 2*L*, *L_y_* = *L*, and *L*_z_ = *L* (see Figure 1) around them. For computational simplicity, we partition such a volume into a cubic lattice of 2*L* ^3^ vertexes, with spacing *d*_0_. The diffusing factors randomly move from one to a nearest neighbor vertex on such a lattice. On each vertex no more than one particle can be present at a given time (see Figure 2).

From a Statistical Mechanics point of view, as each molecule interacts with those on its lattice nearest neighbors with an energy, *E*_0_, the system is characterized by its total energy, i.e., by the following Hamiltonian:

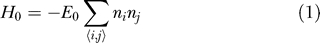
where *E*_0_ is the effective interaction energy, the sum is over all nearest neighbors pairs *i*,*j* on the lattice, and *n_i_* = 0,1 is a variable associated to site *i* corresponding to absence or presence of a molecule. Below we mostly discuss the case where *E*_0_ is in the range of hydrogen bond energies [21]. The X chromosome segments have also an affinity for molecules: each lattice site belonging to the chromosomes has a binding energy *E_X_* (equal for the two X's) with molecules. This corresponds to adding a further term, *H_X_*, to Equation 1:

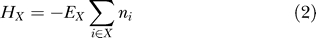
where the sum is on the sites, *i*, belonging to the two X segments. For simplicity, when not differently stated, we take *E_X_* = E_0_. The present SB model [20] is a generalization of the Ising lattice gas model, used in Statistical Physics to describe particles diffusing in a fluid [22].

We investigated by Monte Carlo (MC) computer simulations [23] (see Methods) the dynamics and the final state attained by the present system at a given concentration, *c*, of diffusing particles (below we take *c* = 0.025). At each MC time step, the probability of a particle moving from its location to a neighboring free site is related to the usual Arrhenius factor [23], *r*_0_exp(−Δ*H*/*kT*), where Δ*H* = *H_f_* − *H_i_* is the energy barrier in the move (*H_f_* (respectively, *H_i_*) is the value of *H* = *H*_0_ + *H_X_* in the final (respectively, initial) state, see Figure 2), *k* the Boltzmann constant, and *T* the temperature [21,22]; if there is no barrier to be crossed (i.e., Δ*H* < 0), the probability of the move is proportional to *r*_0_, the bare reaction kinetic rate, related to the ultimate biochemical nature of the molecular factors and of the surrounding viscous fluid. We use *r*_0_ = 30 s^−1^, a typical value in biochemical kinetics, which sets the time unit here. The “random walker” model is recovered when *E*_0_ = 0, i.e., in absence of interaction.

### Self-Assembling of the Blocking Factor

**Figure 3:**
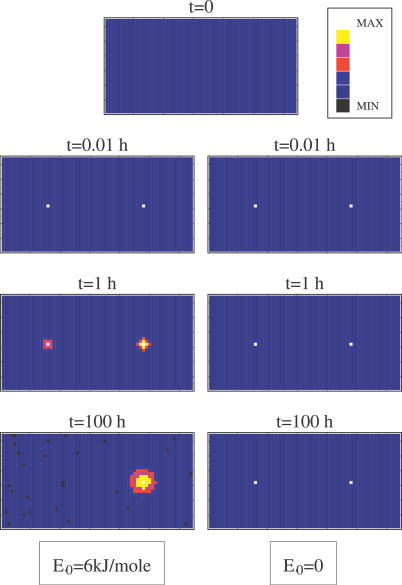
Projection of the Particle Density Distribution on the Plane Orthogonal to the “Chromosomes” at Time *t*, *P*(*x*,*y*;*t*), Shown in a Color Scale during a Single Typical Run (Here the System Linear Size Is *L* = 32*d*_0_ and Molecule Concentration Is *c* = 0.025) In contrast to the “random walk” case *E*_0_ = 0 (right), if *E*_0_ = 6 kJ/mole (left), particles accumulate after a transient around a single “chromosome” as the region around the other one is depleted.

We recorded the probability distribution *P*(*x*,*y*,*z*;*t*) to have a particle at coordinates *x*,*y*,*z* and time *t*, during a given run. In Figure 3 we plot, in a color scale, its projection, *P*(*x*,*y*;*t*), on the *x*-*y* plane (orthogonal to the “chromosomes”) at four characteristic times *t*. *P*(*x*,*y*;*t*) is initially flat since we start from a fully random initial configuration of particles. In the “random walk” case (i.e., *E*_0_ = 0), after a short transient, two small stable peaks are formed in correspondence with the location of the two chromosomes (x_l_,y_l_) = (L/2,L/2) and (x_r_,y_r_) = (3L/2,L/2). In the *E*_0_ = 6 kJ/mole case, at long times it is apparent that one of the early two peaks is going to dominate by far the other (if *P*(*x*,*y*,*z*;*t*) is averaged on all runs, i.e., also on those where a peak on the left is formed, the overall distribution is, of course, symmetric). This shows that, in the “random walk” case, particles diffuse without forming any structure and the configuration at *t* = 0 is almost indistinguishable from the final one except for some binding on each “chromosome.” When *E*_0_ = 6 kJ/mole, the interaction between particles leads to the formation of a single major “complex” [20], as particles tend to aggregate and eventually form a single big cluster binding only one of the chromosomes, as shown by the final state attained by the system in Figure 3.

**Figure 4:**
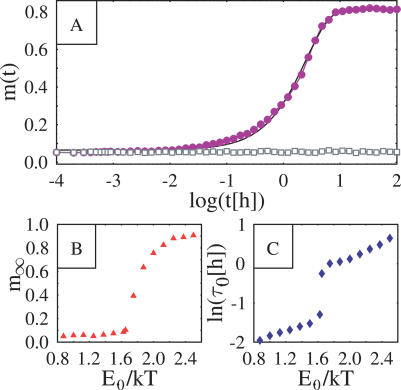
System Plotted as a Function of Time (A), and the Asymptotic Value and the Characteristic Time Scale Plotted as a Function of Particle Interaction Energy (B) (A) System “order parameter,” *m*(*t*) = | *ρ_l_*(*t*) − *ρ_r_*(*t*) | / (*ρ_l_* + *ρ_r_*) (where *ρ_l_* and *ρ_r_* are the average concentration around the “left” and “right” X, see text) is plotted as a function of time in a logarithmic scale (for a concentration *c* = 0.025 of molecular particles). Circles refer to the case *E*_0_ = 6 kJ/mole and squares to *E*_0_ = 0. If *E*_0_ = 0, the symmetry between the two chromosomes is preserved during the evolution and *m*(*t*) = 0. When *E*_0_ = 6 kJ/mole, the symmetry is broken: after a transient, *m* grows one order of magnitude larger than at the starting point. The continuous line is the exponential fit described in the text. (B,C) The asymptotic value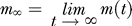 and the characteristic time scale *t*_0_ are plotted as a function of particle interaction energy, *E*_0_ >(normalized by the thermal energy scale *kT*). The drastic change of behaviour around *E*_0_ ∼ 1.6 kT signals the transition to the SB phase (see text).

We stress that only when a precise balance between entropy reduction and energy gain is achieved in the cluster assembling process is a single complex formed, i.e., the symmetry between the X chromosomes is broken. At a given concentration of the particles, *c*, the self-assembly of a single complex only occurs when the interaction energy, *E*_0_, is above a critical threshold value E^*^(*c*) of the order of a weak hydrogen bond [20] (see Figure 4B), which is a decreasing function of *c* and corresponds to a first order phase transition line [22]. In the control parameter plane, (*E*_0_,c), the region where the symmetry is broken extends broadly [20], showing the robustness of the mechanism. As our model can be mapped into the Ising model of Statistical Mechanics, the features of its transitions fall into the Ising universality class [22].

### The Dynamics

Now we turn to the dynamics of the complex formation. Figure 4A shows the evolution, during the same kind of run discussed in Figure 3, of the system “order parameter”, *m*(*t*), defined as *m*(*t*) = | *r_l_*(*t*) + *r_r_*(*t*) | / (*r_l_* + *r_r_*), where *r_l_*(*t*) and *r_r_*(*t*) are the average concentration around the chromosome on the left and on the right at time *t* (*r_l_* = *N_l_* / *V_l_* where *N_l_* (*r_l_* = *N_l_* / *V_l_* where *N_l_* is the number of particles in a cylinder, of radius *R* = 2.5 *d*_0_ and volume *V_l_* = p*R* ^2^ *L*_z_, centered around the left chromosome; analogously, *r_r_* = *N_r_* / *V_r_* is defined). The extent of the SB is clearly illustrated by the equilibrium value reached by *m*(*t*). In the random walk case, *m*(*t*) is fluctuating close to zero, i.e., particles are equally likely to be found around the “left” and “right” X. When *E*_0_ = 6 kJ/mole, *m*(*t*) rises to about 80%, implying that almost all particles are driven into the space around one of the two chromosomes. The time evolution of *m*(*t*) can be approximately fitted, at large *t*, by an exponential function (continuous line in Figure 4A): *m*(*t*) = *m*_∞_ − (*m*_∞_ − *m*_0_)exp(−t / τ_0_), where *m*_0_ and *m*_∞_ are its initial and final values, and τ_0_ the characteristic time scale of the assembling process. The equilibrium value of *m*(*t*), *m*_∞_, and of τ_0_ depends on the three model parameters (*E_X_*,*E*_0_,*c*). The behavior of *m*_∞_, as a function of *E*_0_ is shown in Figure 4B, where the location of the transition point *E* ^*^ is apparent: both *m*_∞_ and τ_0_ have a drastic change of behaviour at *E* ^*^. In particular, *m*_∞_(*E*_0_) is very close to zero (i.e., no major complex is formed, as particles are evenly distributed is space) for *E*_0_ < *E* ^*^, while it becomes definitely larger than zero for *E*_0_ > *E* ^*^ (i.e., a single major complex is formed and attached to one X). The behavior of τ_0_ (see Figure 4C) is also interesting: it is smoothly increasing with the molecules interaction energy, *E*_0_, and has a jump at *E* ^*^. We also found that the lower the affinity of the chromosomes, *E_X_*, the longer such a time scale.

In the phase where the single complex is formed, τ_0_ can be interpreted as the waiting time to have the majority of particles around only one of the X's, i.e., as the characteristic time scale of XCI. The process which results in the aggregation of a single complex has an early stage where molecules tend to bind “sticky” chromosomes (see Figure 3). Later on, a diffusion of molecules from one to the other chromosome takes place, leading to the assembling of one main complex bound to a single X, as the other X remains “naked.” We found [20] that the time, τ_0_, to complete the aggregation of the single complex increases with the square of the X segments distance (as expected in Brownian-like diffusion processes). This suggests that only when the X's colocalize can the complex be assembled in a time short enough to be useful on the cell time scales. For instance, if the average distance between X's without colocalization is a factor of 10 larger, the assembling time is increased by two orders of magnitude, which is far longer than the cell cycle itself.

Summarizing, in the SB model, the molecular factor interaction induces formation of clusters; if *E*_0_ is above a given threshold, *E* ^*^, a thermodynamics phase transition occurs and clusters eventually coalesce in a single major “complex” [20]. The self-assembly of the latter can explain the spontaneous formation of a single BF complex in XCI and its time scale the importance of X colocalization.

### Blocking Factor Binding to X Chromosomes

We now turn to the role in our model of *E_X_*, i.e., the chemical affinity of the multiple binding sites of X segments for the molecular factors eventually forming the BF. We investigate the effects of changes of *E_X_* and the role of an affinity gap, Δ*E*, between the two chromosomes. This kind of analysis is important to rationalize and to predict how mutations of the X segments relevant to BF binding, such as deletions, transgenic insertions, or chemical modifications, may affect the efficiency and, in general, the outcome of XCI. As mutations result in a change of the overall affinity of X segments, within our schematic model we can understand, in particular, their effects on the binding probability of the BF to the X's. So, we consider now the more general situation where the binding energies to the “left” and “right” chromosomes, *E_Xleft_* and *E_Xright_*, can be different. We set *E_Xleft_* equal to a given value *E_X_*, and introduce on *E_Xright_* a relative energy skewing, Δ*E*: *E_Xright_* = (1 − Δ*E*)*E_X_*.

The binding of the BF results in a relative abundance of molecular factors around the “left” or “right” X, much larger than expected by a random fluctuation. So, in the phase where the BF is formed, after measuring the average concentration of molecules around the “left”, *r_l_*(*t*), and “right”, *r_r_*(*t*), chromosomes, we can check if and where the BF is bound at time *t*. To this aim, we compute the ratio R = [*r_l_* (*t*) − *r_r_* (*t*)] / [*r_l_* (*t*) + *r_r_* (*t*)]. Whereas *R* is zero in a system where particles are randomly distributed, the attachment of molecular factors preferentially to one chromosome induces a skewing in *R* (as much as in *m* above). We consider the BF bound to the left (respectively, right) chromosome if *R* > 50% (respectively, <−50%), otherwise it is considered unbound (the 50% threshold used here is not important in itself, as any other large enough value would do). We can then measure the average probability for the BF to be bound, at time *t*, to the “left” or the “right” X, *p_l_*(*t*) and *p_r_*(*t*), or to be unbound, *p_u_*(*t*) (with *p_l_* + *p_r_* + *p_u_* = 1). These quantities give direct access to important information on XCI establishment (and cell survival): for instance, we can measure the probability that the BF misses its target sites on the X chromosomes, or investigate skewing in random XCI induced by a gap between *E_Xleft_* and *E_Xright_*.

**Figure 5:**
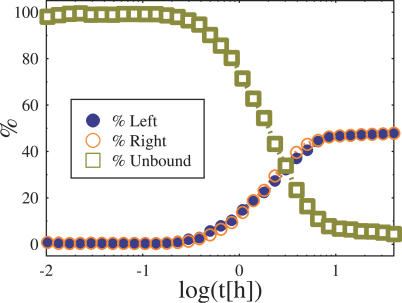
Probability Plot of the Blocking Factor Complex to Bind, at Time *t*, the “Left” and “Right” Chromosome, *p_l_*(*t*) (Filled Circles), and *p_r_*(*t*) (Empty Circles), and the Probability To Be Unbound, *p_u_*(*t*) (Squares) The case shown here is for *E_x_* = 4*kT*, Δ*E* = 0, *E*_0_ = 2.4 *kT*, and *c* = 0.025.

We first discuss the case where Δ*E* = 0, i.e., there is no skewing between the two X segments. The time evolution of *p_l_*(*t*), *p_r_*(*t*), and *p_u_*(*t*), and in the simulations previously described, is plotted in Figure 5 where we illustrate the case with *E_X_* = 4 *kt* and ΔE = 0 (*E*_0_ = 2.4 *kT* and *c* = 25 × 10^−2^). Initially, we have *p_u_*(_0_) = 1 and *p_l_*(0) = *p_r_*(0) = 0. From Figure 5 we see that, after an early transient of the order of a few hours, *p_u_*, *p_l_*, and *p_r_* saturate to their asymptotic value: the binding probabilities *p_l_* and *p_r_* grow in time *p_l_*(*t*) = *p_r_*(*t*) (at all times, *t*, since Δ*E* = 0), conversely *p_u_*(*t*), after the transient drastically decreases well below 1. Importantly, *p_u_* is still not zero even after about 20 h from the starting configuration, implying that there is a finite chance for the BF to miss its target within scales of the order of the cell cycle, leading to failure of XCI. This result points out that even in a normal population of cells a fraction is bound to die, a fact observed in experiments.

**Figure 6:**
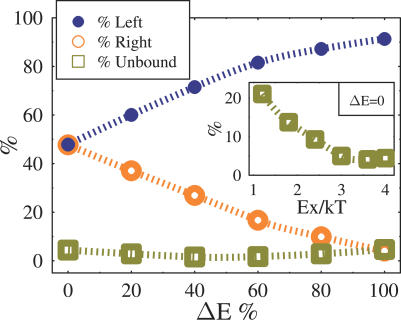
Value of Probabilities (from Figure 5) for the BF To Bind the “Left” or “Right” X, *p_l_* (Filled Circles) and *p_r_* (Empty Circles), and To Be Unbound, *p_u_* (Squares), Are Plotted After 20 Hours (i.e., After the Transient Regime from the Initial Configuration) as a Function of the Relative Skewing Energy between the “Chromosomes,” Δ*E* Here *E_x_* = 4*kT*, *E*_0_ = 2.4 *kT*, and *c* = 0.025. **The inset** shows *p_u_* as a function of *E_x_* when Δ*E* = 0 (here *p_l_* = *p_r_* = (1 − *p_u_*) / 2). Interestingly, *p_u_* is nonzero also for comparatively high values of *E_x_*.

The long time values of *p_u_*, *p_l_*, and *p_r_* depend on the parameters *E_X_* and Δ*E*. In particular, we describe now how these parameters affect the values of *p_u_*, *p_l_*, and *p_r_* recorded at *t* = 20 h, a biologically relevant time scale of the order of the cell cycle, located well after the transient mentioned above. The inset of Figure 6 plots *p_u_*(t = 20 h) as a function of *E_X_* for Δ*E* = 0. Interestingly, *p_u_*(t = 20 h) decreases with *E_X_*, but it is still nonzero for comparatively high *E_X_* (in the case shown, it is still above 5% when *E_X_* = 4 *kT*); when *E_X_* is reduced, *p_u_* becomes markedly different from zero (e.g., in the case shown, *p_u_*(t = 20 h)20% for *E_X_* = 1 *kT*), resulting in a significant fraction of cases where a proper XCI fails, since both chromosomes can be unprotected from inactivation. In fact, the above remarks can explain in a quantitative way experimentally observed failure of XCI in homozygous deletions (see next section).

### XCI Skewing

We now discuss the case where a non zero gap, Δ*E*, is present between the affinities of the X chromosomes. The values of *p_u_*, *p_l_*, and *p_r_* after 20 h are plotted in Figure 6 as a function of Δ*E* (in the case *E_X_* = 4 *kT*). As expected, when Δ*E* increases, *p_r_* decreases and eventually, for Δ*E* = 100%, approaches *p_u_*, the “background” value. These findings can explain on a quantitative ground the skewing of XCI observed in heterozygous deletion experiments (see next section): an energy gap Δ*E* between *E_Xleft_* and *E_Xright_* can result in a dramatic difference in the binding probabilities of the BF on the two X chromosomes. More specifically, the present model opens the way to an experimental measure of the overall binding energies *E_Xleft_* and *E_Xrigh_*, which can be derived by a combined measure of experimentally accessible quantities such as *p_u_* and *p_l_*.

## Discussion

The SB model describes the self-assembling of a single controlling complex, biologically interpreted as the “BF” in XCI, that attaches to one of the two X segments, designating the active X, as it is the only one to be protected by a dense enough coating of molecules [20]. Similarly, one can show that when more than two X's are present in the nucleus the factors end up binding to only one, explaining why in diploid cells with X chromosome aneuploidy only one active X is found. In male cells the only X present is consistently protected from inactivation because it has no competitors in binding the blocking complex.

We showed that even a small difference in the values of the affinities, *E_X_*, of the X chromosomes for the BF, e.g., induced by chemical or physical modifications, skews particle binding so that the complex is usually assembled on the chromosome with higher affinity. This result can explain why allelic differences in *Xic* sequences, such as in the *Xce* locus [24] or other regions [2,3,8], give rise to biased X-inactivation.

We now discuss important experiments on “counting” and “choice” which consider deletions on X chromosomes or transgenic insertions into autosomes. In our perspective, a deletion in a segment, including binding sites for the molecular components of the BF, results in the reduction of the chemical affinity of such a sequence for the BF; analogously, the insertion of a similar segment into an autosome results in the possibility of the autosome to bind the BF. So, with reference to the data reported in the previous section, we can interpret, on a quantitative ground, deletion and insertion experiments. We first consider an important deletion which was instrumental in defining the role of the region 3′ to *Xist* in counting and choice, namely Δ65 kb [10]. The Δ65 kb deletion removes 65 kb of DNA in the *Xic* region relevant to the chromosome activation [10] spaning the 3′ exons of *Xist*, the 5′ of *Tsix*, and *Xite*. We also discuss a series of nested subdeletions [25] and addbacks [26] within the Δ65 kb and the *Tsix* ^Δ*CpG*^ deletion [11] removing a small, 4 kb, sequence from the *Tsix* promoter.

Δ65 kb causes nonrandom inactivation of the deleted X in heterozygous XX cells [10], and *Tsix* ^Δ*Cp*^ [11] has an analogous effect. This is interpreted in usual BF models by assuming that such a deletion removes the binding site for the BF. In particular, our model describes, in a quantitative way, how such an effect is related to Δ*E*, i.e., to the nature of the deletion, as illustrated by the results of Figure 6: the mutated X, having a reduced overall affinity for the BF, i.e., a difference in energy Δ*E* with respect to the other X, loses on average the competition for the complex. This, in turn, leads to a skewing in XCI, with the mutated X being inactivated more frequently than the other X.

The X chromosome bearing the Δ65 kb deletion is not active in (XY) male cells as well. Importantly, other shorter deletions nested into the Δ65 kb have been described that cause ectopic X inactivation in male cells. The Δ*AS* deletion [25] causes a minimal, but detectable, level of ectopic X inactivation. A 1.2 kb deletion of *DXPas*34, spanning an array of CTCF binding sites also implicated in choice (see also [27,28]), causes XCI to initiate in a significant proportion of male cells [25]. Finally, the Δ*AV* deletion [25], extending 3′ to *Xist* to include *DXPas*34 and the major *Tsix* promoter, causes ectopic X inactivation in male cells with high efficiency. These mutations also suggested that similar mechanisms regulate XCI, or lack thereof, in both female and male cells [25].

The effect of these short deletions, unexplained by usual BF models, can be simply understood within the SB model framework, which can explain, as well, their “probabilistic” character, where only a fraction of cells initiate ectopic XCI. As the Y chromosome doesn't bind the BF, in our schematic model it can be described as an “X” with Δ*E* = 100%. The mutated X chromosome, in turn, has a reduction in its overall binding energy for the BF if some of its binding sites have been deleted: schematically, the smaller the nested deletion within the Δ65 kb the weaker the reduction is expected to be. Such a situation (with Δ*E* = 100% and a reduced *E_X_*) is analogous to the one depicted in Figure 6 and its inset, and gives rise to very similar results, such as an increase of *p_u_* (i.e., the probability of the BF to be unbound) when *E_X_* is decreased. So, we can understand that in two populations, with either a short nested or long mutation, the fraction of cases where the BF won't bind the X, leading to failure of proper XCI, can be quite different as they have very different values of *E_X_* (if the deletion is too long, as in the Δ65 kb deletion, it can even be unable to bind the BF). The observation that in male cells the *Tsix* ^Δ*CpG*^ deleted X remains active [11] could have an analogous explanation.

The analysis of reinsertions into the Δ65 kb deletion, in heterozygous female cells, provides striking evidence that choice can be dissected away from its likely downstream effector mechanism [26]. While the Δ65 kb deletion causes an increase in the amount of *Xist* RNA produced from the deleted chromosome in both undifferentiated and differentiated ES cells, the reinsertion of 16 kb (3′ to *Xist* up to include *Tsix* initiation site) is able to restore normal levels of *Xist* expression, and yet it does not restore random choice, the deleted chromosome being chosen for inactivation [26]. As discussed above, these experimental results can be explained within the SB scenario where XCI skewing can be obtained by deletions of a fraction of BF multiple binding sites.

In homozygous *Tsix* ^Δ*CpG*^ XX mutants (i.e., female cells with both X's mutated), the choice of the active X is still random [17], but, importantly, in a fraction of cells both X's are inactivated (“chaotic counting” [8]). This is another result that usual BF models cannot explain: if the deleted region does not include the BF binding site, only one inactive X should be found, whereas two inactive X should always be seen if it does [8]. The data from the SB model shown in the inset of Figure 6 allows us to understand the issue: if the affinity *E_X_* for the BF is reduced, the X's succeed in binding the BF in a smaller fraction of cases, increasing the chances of failure of XCI. In the case of a much longer deletion (say Δ65 kb) the X's would be unable to bind BF and, thus, are both inactivated.

Transgene insertions into autosomes have also been analyzed [9,29,30]. When long *Xic* transgenes are introduced, in multiple copies [29], into autosomes of male ES cells, inactivation of the single X occurs in a fraction of the cells [9,30]. The frequency of inactive X chromosomes, in different male transgenic lines, increases with increasing number of copies of the transgene [9]. In this case the transgenic autosome can have a finite affinity for the BF, although a gap Δ*E* can be present with respect to the X chromosome. From the results of Figure 6, our model predicts indeed that the X is wrongly inactivated in a fraction of cases, since it can lose the BF to the transgenic autosome.

The BF model, focusing on the mechanisms designating the active X chromosome, could represent only some aspects of “counting and choice” in XCI. For instance, some experimental results suggest that cis-acting regulatory sequences in the *Xic* could concur in designating the inactive X [16,31,32]. To explain these observations, a two-factor model has been proposed which postulates that a unique BF and a nonlimiting “initiator” factor exist, displaying mutually exclusive binding to the two X chromosomes [16]. In such an extended scenario, the occurrence of a single BF per cell could be explained, as well, by the SB mechanism.

The chain of events that follow the binding of the BF has not been determined yet and multiple layers of regulation may contribute to choice. A possible hypothesis is that the BF upregulates *Tsix* transcription, which in turn represses *Xist*, and, thus, allows the BF-binding chromosome to remain active (see [2–4,25] and references therein). For instance, a repressive effect of *Tsix* in cis on *Xist* expression has been documented in [26,33,34]. Truncation of *Tsix* transcription causes the mutated X to be chosen as the inactive chromosome [33,34], suggesting an in-cis role for the *Tsix* transcript in choice. It has been speculated that transcription of *Xist* or *Tsix* causes changes in affinity of the mutated chromosome for regulatory factors as a consequence of chromatin remodeling [35].

Finally, the SB mechanism shows that there is a typical “time scale” for the protection of the active X to occur, as the supermolecular BF takes some time to bind and grow on a randomly designated chromosome. At intermediate time points, factors accumulate on both chromosomes (see Figure 3); later on, one cluster prevails over the others. When the initial concentration, *c*, of autosomal factors is very high (e.g., double in tetraploid cells) the clusters formed at intermediate stages are larger in size (e.g., doubled), and may behave as “BFs.” This can explain the stochastic nature of the X chromosome/autosome ratio effect [2–4]. The SB model also reveals that the proximity of the *Xic* on the colocalized chromosomes enhances the probability of the formation of a single BF complex in an appropriate time, by increasing, e.g., the chance that a cluster detaches from one chromosome and is “captured” by the prevailing other. Actually, the time, t_0_, to form the final complex rapidly grows with the X segment distance, *L*, explaining the important role of X colocalization [20].

Summarizing, in this study we devised a physical mechanism (illustrated via a schematic SB model) for the self-assembling of a single supermolecular complex which spontaneously breaks the binding symmetry of two equivalent targets. This embodies a new stochastic regulatory mechanism resulting from collective behavior at a molecular level.

In the SB model scenario, we explained by quantitative simulations how an X-inactivation theory based on a supermolecular controlling complex can physically work, independently from its ultimate biochemical details. It ascribes the features of random X inactivation to the mechanism of assembly and binding of the BF. In the present view, the BF is a cluster of transacting factors which can bind many a site on a chromosome at the same time and coat, in particular, a region regulating (directly or indirectly) *Xist* expression. We showed on a thermodynamic ground how the blocking complex is self-assembled from many diffusible molecules, and why only one is formed (many autosomal molecules could produce many a complex), i.e., the symmetry of X chromosomes is broken. Finally, we discussed the binding properties of the BF to the X and, in particular, the situation where the X chromosomes are mutated. For sake of clarity, we considered only one kind of soluble factors and a single kind of BF complex, though the model could easily accommodate more than one.

A comprehensive scenario emerges from our SB schematic model, explaining the variety of experiments using deletions or transgenic insertions to investigate “counting and choice” in XCI. Further evidence supporting our picture is found in recent papers [27,28] where candidates for the molecular trans-acting factors have been identified and shown to complex through specific protein–protein interactions. They were also found to bind at several contiguous sites on *Tsix*, an XCI controlling region 3′ to *Xist*, and to be a key component in XCI regulation.

The SB regulatory mechanism, discussed here for XCI, relies on a switch that has a thermodynamics origin, a phase transition occurring in the system [20]. It is, thus, simple and robust enough to be likely to be present in other cell processes, such as cases of monoallelic random expression [6,7].

## Materials and Methods

### Monte Carlo simulations.

In our simulations, particles start from a random initial configuration in the space (see Figure 1) and then diffuse, at room temperature, according to Monte Carlo dynamics of the lattice model descibed above. We considered lattice sizes from *L* = 16*d*_0_ up to *L* = 128*d*_0_ in order to check that our results are robust to size changes. We use periodic boundary conditions, and the averages shown below are over up to 1,024 runs from different initial configurations. Monte Carlo step unit is a lattice sweep [23].

## Abbreviations

BF: blocking factor
SB: symmetry breaking
XCI: X chromosome inactivation
*Xic,*: X-chromosome–inactivation center
*Xist,*: X inactive-specific transcript
